# The Assessment of Anthropometric Measures and Changes in Selected Biochemical Parameters in Obese Children in Relation to Blood Lead Level

**DOI:** 10.3390/metabo14100540

**Published:** 2024-10-09

**Authors:** Katarzyna Pozorska, Irena Baranowska-Bosiacka, Dominika Raducha, Patrycja Kupnicka, Mateusz Bosiacki, Beata Bosiacka, Justyna Szmit-Domagalska, Joanna Ratajczak, Anita Horodnicka-Józwa, Mieczysław Walczak, Dariusz Chlubek, Elżbieta Petriczko

**Affiliations:** 1 Department of Pediatrics, Endocrinology, Diabetology, Metabolic Disorders and Cardiology of Developmental Age, Pomeranian Medical University, 71-252 Szczecin, Poland; katarzyna.pozorska@pum.edu.pl (K.P.); dominika.raducha@pum.edu.pl (D.R.); justyna.szmit.domagalska@pum.edu.pl (J.S.-D.); anita.horodnicka.jozwa@pum.edu.pl (A.H.-J.); mieczyslaw.walczak@pum.edu.pl (M.W.); elzbieta.petriczko@pum.edu.pl (E.P.); 2 Department of Biochemistry and Medical Chemistry, Pomeranian Medical University, 70-111 Szczecin, Poland; patrycja.kupnicka@pum.edu.pl (P.K.); mateusz.bosiacki@pum.edu.pl (M.B.); dchlubek@pum.edu.pl (D.C.); 3 Institute of Marine and Environmental Sciences, University of Szczecin, 71-415 Szczecin, Poland; beata.bosiacka@usz.edu.pl; 4 Institute of Physical Culture Sciences, University of Szczecin, 71-065 Szczecin, Poland; joanna.ratajczak@usz.edu.pl

**Keywords:** lead (Pb), blood lead level (BLL), children obesity, children overweight

## Abstract

Background: Our paper draws attention to the impact of lead (Pb) on the specificity of obesity development in children exposed to environmental pollution. An advantage of this paper is the homogeneous study group comprising children of identical age from a single geographic region. Moreover, while the influence of environmental toxins on adults has been extensively explored, this study delves into pediatric populations, which have yet to receive comprehensive scrutiny within the scientific literature. Methods: Initially, a group of 136 obese children (the research program lasted three consecutive years: 2016, 2017, and 2018) living in the north-western region of Poland, from whom biochemical tests and auxological data were obtained, were enrolled for analysis. Blood lead levels (BLLs) were determined in 115 children. The age of the children ranged from 7.1 to 10.4 years. The body mass index (BMI) of children averaged 21.5 ± 2.2. Results: The results showed that a large proportion of the participants had BLLs above the threshold for Pb. BLLs ≤ 5 µg/dL (considered safe for children and pregnant women) were found in over 70% of the participants, with BLLs in the range of 5.01–10.00 µg/dL in over 26% of the children, and concentrations > 10 µg/dL (considered toxic threshold for adults) in nearly 2% of the children. The results of our research revealed a positive association between BLLs and average systolic and diastolic blood pressure in the studied children. Moreover, we found a negative correlation between BLLs and absolute fat tissue content and triglyceride concentration. Among the included biochemical factors, only insulin demonstrated a statistically significant relationship with fat mass. This result suggests that early carbohydrate metabolism disorders in overweight children involve decreased peripheral tissue insulin sensitivity. Conclusions: Lead exposure may significantly contribute to the development of hypertension, insulin resistance, and glucose metabolism disorders in overweight and obese children. It is essential to implement multidirectional actions to increase awareness of the harmful effects of xenobiotic exposure, including lead, in order to prevent early-life exposure.

## 1. Introduction

Obesity, a chronic metabolic disorder, is on the rise among adults and children in highly developed countries and has been designated as a 21st-century epidemic by the World Health Organization (WHO). It is estimated that approximately 650 million people worldwide are affected by obesity, with 23.2% of the population in Poland suffering from this condition [1]. Diagnosing and assessing obesity in adults rely mainly on the body mass index (BMI), defined as body mass (kg) divided by height squared (m^2^), with a BMI of ≥30.0 kg/m^2^ indicating obesity [2].

Traditionally, obesity was attributed to an imbalance between energy intake and expenditure, resulting in the accumulation of adipose tissue. However, contemporary discourse among scientists challenges this view, highlighting the role of environmental toxins, food additives, and the interaction between genetic and environmental factors [3]. Of particular interest are obesogens, exogenous substances that disrupt lipid metabolism, appetite and satiety regulation, and energy balance, thereby increasing the risk of obesity [4].

One potential obesogen that may be ubiquitous in the environment is lead (Pb), an endocrine disruptor that significantly affects hormonal balance [5]. It ranks as the second most hazardous environmental toxin [6]. Although a safe Pb concentration threshold remains undetermined, it is estimated to contribute 0.6% to the “global burden of disease” [7].

Childhood Pb exposure in developed countries typically involves chronic low-level exposure, termed microintoxication, which leads to metabolic and neurological disturbances. Recent studies reveal adverse effects at levels lower than those once considered safe (i.e., below 10 μg/dL), prompting a revised limit of 5 μg/dL in whole blood (blood lead level—BLL) for children and pregnant women [8,9,10,11]. It has been proven that both prenatal and postnatal exposure to Pb leads to an increased risk of metabolic and structural changes in the development of the central nervous system. Children exposed to Pb in the early stages of development develop a number of neurological disorders, such as motor hyperreactivity, concentration disorders, autism spectrum symptoms, and tendencies to lower IQ. It is now known that at levels lower than 10 μg/dL in whole blood, exposure to Pb can also lead to neurobehavioral disorders and lower IQ [10,11].

In October 2021, the Centers for Disease Control and Prevention (CDC) updated the Blood Lead Reference Value (BLRV) from 5.0 μg/dL to 3.5 μg/dL, aiming to identify children with elevated BLLs [12]. The value is based on the 97.5th percentile of blood Pb distribution in children aged 1–5 years in the USA. By updating BLRV to 3.5 μg/dL, children with BLLs in the 3.5–5 μg/dL range can now also receive prompt help to mitigate health effects and remove or control sources of exposure. However, BLRV is not a health standard or toxicity threshold; it should be used as a guideline to help determine if further medical or environmental testing is recommended, and to prioritize communities most in need of primary exposure prevention. It should be emphasized that the BLL of 5 µg/dL is a threshold for taking action to reduce further exposure. Any amount of Pb exposure can be harmful especially for newborns who do not yet have a fully formed blood–brain barrier [12].

Society remains largely unaware of the long-term problems related to chronic contamination with Pb and other metals. Therefore, this paper draws attention to the impact of Pb on the development of obesity in children exposed to environmental pollution. An advantage of this paper is the homogeneous study group comprising children of identical age from a single geographic region. Moreover, while the influence of environmental toxins on adults has been extensively explored, this study delves into pediatric populations, which have yet to receive comprehensive scrutiny within the scientific literature.

## 2. Materials and Methods

### 2.1. Study Population

This study was conducted on a cohort of 136 children residing in the West Pomeranian Voivodeship. The participants were part of the “Courageous Eight” program, which was designed to address weight management issues among school-aged children in Szczecin, Poland. The program was a collaborative effort between the Independent Public Clinical Hospital No. 1 of the Pomeranian Medical University in Szczecin, the University of Szczecin, and the Non-Public Health Care Institution “School Medicine” (“SZKOLMED”) in Szczecin, Poland.

The program spanned three consecutive years (2016, 2017, and 2018) and focused on children born in specific years: 2008, 2009, and 2010, respectively. Funding for the program was provided by the Municipality of Szczecin, Poland and it was developed under the auspices of the Polish Society of Health Programs in Gdańsk, Poland. The Society also conducted an evaluation of the program and served as an independent auditor for the project.

### 2.2. Initial Phase of the Program

The program’s initial phase involved screening and conducting a comprehensive health evaluation of all eight- and nine-year-old children in Szczecin, Poland (those in second and third grades) whose parents consented to participation. The screenings took place in all primary schools in Szczecin, Poland including public, private, and special schools, where a team of trained school nurses performed the assessments. They carefully logged all patient information and test results into a custom computer program designed for the project.

During this initial phase, the examinations included measurements of basic anthropometric parameters like body height, weight, BMI, waist and hip circumference, as well as assessments of arterial blood pressure and body composition. Children with excessive body weight, identified by a BMI ≥ 90th percentile, were then advanced to the next phase of the program. Results were compared against Polish growth charts from OLAF and OLA studies conducted between 2007 and 2012, with criteria set by the CDC—BMI ≥ 85th–95th percentile for overweight and BMI ≥ 95th percentile for obesity [13,14]. The OLAF study “Obesity and arterial hypertension in adolescents and their chronic complications” focused on examining the prevalence of obesity and hypertension among adolescents in Poland and the potential chronic complications associated with these conditions. The OLA study “Obesity and arterial hypertension in Lublin, Poland and surrounding areas” specifically targeted the region of Lublin and its surrounding areas to investigate the prevalence of obesity and hypertension among residents in this area.

Additionally, during the first project visit, blood lead levels (BLLs) were measured in 115 children as an extra assessment to evaluate potential environmental influences on health. Unfortunately, not all parents and guardians provided consent for additional blood sample collection.

### 2.3. Data Collection Procedure

#### 2.3.1. Anthropometric Measurements

During both visits, standardized procedures were followed to assess anthropometric parameters. Height measurements were taken using a Harpenden stadiometer in the Frankfurt position, with a precision of 0.1 cm. Body weight was assessed using a body composition analyzer with an accuracy of 0.1 kg. Waist and hip circumferences were measured using a calibrated tape with a precision of 0.1 cm. To compare the result of a single observation of height and body mass with population data, height was expressed as SDS (standard deviation score) using the formula: $\mathrm{HSDS}=\frac{h\left[\mathrm{cm}\right]-h_{50c}\left[\mathrm{cm}\right]}{0.5\times \left(h_{50c}\left[\mathrm{cm}\right]-h_{3c}\left[\mathrm{cm}\right]\right)}$, where *h*—body height; *h*_50*c*_—body height for the 50th percentile; *h*_3*c*_—body height for the 3rd percentile. Body weight measurements were taken using a medical scale. The BMI was calculated using the formula: $\mathrm{BMI}=\frac{b.w.\left[\mathrm{kg}\right]}{ht[m2]}$, where *b.w.*—body weight; *ht*—body height.

The results obtained were compared to norms specific to the Polish population. For statistical analysis, height and body weight, as well as BMI, were expressed as SDS relative to the norm for sex and age, using the formula: b.w. $\mathrm{SDS}=\frac{b.w.\left[\mathrm{kg}\right]-b.w._{50c}\left[\mathrm{kg}\right]}{0.5\times \left(b.w._{50c}\left[\mathrm{kg}\right]-b.w._{3c}\left[\mathrm{kg}\right]\right)}$, where *b.w.*—body weight; *b.w.*_50*c*_—body weight for the 50th percentile; *b.w.*_3*c*_—body weight for the 3rd percentile [15].

#### 2.3.2. Blood Pressure

Before blood pressure measurements, participants underwent a 5 min resting period in a calm environment, seated on a chair with back support, legs uncrossed, and feet flat on the floor. They refrained from physical activity for at least 30 min before the examination. Blood pressure was then measured three times using an Omron 2 electronic blood pressure monitor with an appropriately sized cuff, placed directly on the patient’s arm without any clothing obstruction. The arm was supported to ensure the cuff’s center aligned with the level of the right atrium. No conversations took place during the measurement process. Blood pressure assessments were consistently carried out during the screening phase and at each of the four intervention visits (4 visits during 12 months) [16].

#### 2.3.3. Body Composition Analysis

During the screening phase, body composition analysis was performed using a Jawon Medical X-Contact 350 body composition analyzer. In the intervention stage, a Jawon Medical IOI 353 was used with the electrical bioimpedance (BIA) method. The analysis included determining parameters such as the percentage of body fat [kg], fat mass [kg], lean body mass [kg], water mass [kg], and muscle mass [kg].

### 2.4. Data Analysis Methods

#### 2.4.1. Biochemical Analyses

Venous blood samples were collected in the morning after an overnight fast and rest using vacuum tubes. A comprehensive analysis was performed, including determining concentrations of glucose, insulin, lipid profile (total cholesterol, LDL (low-density lipoprotein) and HDL high-density cholesterol), triglycerides, as well as TSH (thyroid-stimulating hormone), fT4 (thyroxine), aspartate aminotransferase (AST), and alanine aminotransferase (ALT). Laboratory tests were conducted twice during the program for a thorough evaluation. The following methods were utilized:

- Glucose concentration: the enzymatic method with hexokinase;
- Insulin concentration: the electrochemiluminescence method (ECLIA);
- Total cholesterol concentration: the enzymatic colorimetric method;
- HDL and LDL cholesterol concentrations: the homogeneous colorimetric enzymatic method;
- Triglycerides (TG) concentration: the enzymatic colorimetric method;
- TSH concentration: electrochemiluminescence assay (ECLIA);
- fT4 concentration: electrochemiluminescence assay (ECLIA);
- AST concentration: the kinetic method;
- ALT concentration: the kinetic method.

All analyses were performed using the Cobas PRO c503 module by Roche Diagnostics. Reference values were 60–99 mg/dL for glucose, 2.6–24.9 μIU/mL for insulin, and ALT 0–44 U/L and AST 0–50 U/L for liver enzymes. Reference values for lipids are detailed in Table 1.

Based on the obtained results, the HOMA-IR index (Homeostatic Model Assessment for Insulin Resistance) was calculated after both the first and fourth visits using the formula HOMA − IR = I0 × G0/22.5, where I0—fasting insulin [μIU/mL] and G0—fasting glycemia [mg/dL].

#### 2.4.2. Lead Determination

Lead in whole blood was analyzed using inductively coupled plasma optical emission spectrometry (ICP-OES, ICAP 7400 Duo, Thermo Fisher Scientific, Poznań, Poland), employing a spectrometer equipped with a concentric nebulizer and cyclonic spray chamber, and operated in the axial mode. For digestion, the samples were treated using a microwave digestion system, specifically the MARS 5 from CEM Corporation, Matthews, NC, USA. Each blood sample (1 mL) was placed into clean polypropylene tubes, to which 1 mL of 65% HNO_3_ was added. After allowing a 30 min pre-reaction time in a clean hood, 1 mL of non-stabilized 30% H_2_O_2_ solution was introduced to each vial. Subsequently, the samples were transferred to special Teflon vessels and subjected to heating in the microwave digestion system for 35 min at 180 °C (15 min ramp to 180 °C and maintained at 180 °C for 20 min). Following digestion, the samples were cooled to room temperature.

Post-digestion, the samples were transferred to acid-washed 15 mL polypropylene tubes in a clean hood and stored in a monitored refrigerator at approximately 4 °C until analysis.

Prior to ICP-OES measurement, a further 5-fold dilution was carried out. The samples were spiked with an internal standard to achieve a final concentration of 0.5 mg/L Yttrium, and 1 mL of 1% Triton (Triton X-100, Sigma-Aldrich, Poznań, Poland) was added before dilution to a final volume of 6 mL with 0.075% nitric acid (Suprapur, Merck, Poznań, Poland). Blank samples were prepared by adding concentrated nitric acid to tubes without samples, followed by the same dilution process as described above.

Multielement calibration standards (ICP multielement standard solution IV, Merck) were prepared with varying concentrations of inorganic elements, following the same procedures as blanks and samples. Deionized water (Direct Q UV, Millipore, Warsaw, Poland) approximately 18.0 MΩ was utilized for the preparation of all solutions. The wavelength used for Pb measurement was 220.353 nm.

### 2.5. Statistical Analysis Methods

The statistical analysis was carried out using Stata 11 (license number 30110532736) and MS Office Excel (2007). Normality distribution checks were performed on continuous variables using the Kolmogorov–Smirnov test. These variables were summarized with mean values, standard deviations, standard errors, as well as minimum and maximum values.

As the majority of variables displayed normal distributions, and in cases of deviations, the discrepancy (Dmax) was minimal, and the sample size (N) was high, continuous variables were compared using Student’s *t*-test or ANOVA MANOVA. The presentation of variables included mean values, standard deviations, standard errors, and minimum and maximum values.

The results were interpreted using the correlation coefficient (r) and probability (p). Pearson’s correlation was employed to assess the correlation between qualitative and quantitative variables. Statistically significant differences were determined when the probability (p) was equal to or less than 0.05. A significance level of *p* = 0.051–0.099 was considered a trend approaching statistical significance.

To explore the impact of biochemical analysis results (glucose and insulin concentrations, lipid profile including total cholesterol, LDL and HDL cholesterol fractions, triglycerides, as well as TSH, fT4, AST, and ALT) on the body composition of the subjects (percentage of body fat, fat mass, lean body mass, water mass, and muscle mass), a stepwise backward multiple regression analysis was conducted.

The regression model was built with the studied body components as dependent variables and ALT, AST, glucose, insulin, total cholesterol, HDL, LDL, triglycerides, and Pb as independent variables. Not all explanatory variables correlated with the dependent variables in the regression analysis equation were statistically significant. A backward stepwise multiple regression analysis was utilized to illustrate the cause-and-effect relationships between the variables in the equation, providing a comprehensive assessment of the influence of explanatory variables on the formation of body composition components.

## 3. Results

### 3.1. Characteristics of the Study Group

Initially, a group of 136 children, from whom biochemical tests and auxological data were obtained, were enrolled for analysis. Blood lead levels were successfully determined in 115 children. In the study group, there were 80 girls (58.8%) and 56 boys (41.2%) diagnosed with overweight. The age of the children in this group ranged from 7.1 to 10.4 years, with a mean of 8.3 ± 0.7 years. The height of the children in the study group averaged 133.3 ± 6.6 cm, with height SDS ranging from −3.38 to 3.4, and a mean of −0.8 ± 1.1. The weight of the children in the study group ranged from 25.5 to 59.6 kg, with a mean of 38.5 ± 6.2 kg, while weight SDS ranged from 0.3 to 18.6, with a mean of 3.9 ± 2.2. The BMI in the study group averaged 21.5 ± 2.2 kg/m^2^, with an SDS BMI mean of −4.5 ± 1.8. The heights of the parents could not be determined in the entire study group. Maternal weight was assessed in 132 children from the study group, accounting for 97.1% of the entire group. It ranged from 48 to 120 kg, with a mean of 75.5 ± 15.8 kg, expressed as SDS with a mean of 3.7 ± 3.2 and ranging from −1.8 to 12.6. Maternal BMI averaged 27.2 ± 5.5 kg/m^2^. Paternal weight was assessed in 113 children from the study group, accounting for 83.1% of the entire group. It ranged from 60 to 140 kg, with a mean of 93.5 ± 15.3 kg, expressed as SDS with a mean of 3.4 ± 2.1 and ranging from −1.2 to 9.9. Paternal BMI averaged 29.2 ± 4.3 kg/m^2^. Auxological data of the study group are presented in the tables below (Table 2, Table 3, Table 4 and Table 5).

### 3.2. Percentage Distribution of the Analyzed Parameters

Out of 136 participants in this study, 56 were boys, comprising 41.2% of the total group, and 80 were girls, accounting for 58.8% of the study cohort. A BLL of ≤5 µg/dL was found in 83 individuals, constituting 72.2% of the group, while concentrations in the range of 5.01–10.00 µg/dL were observed in 30 individuals, representing 26.1% of the cohort. Concentrations exceeding 10 µg/dL were detected in only two individuals, corresponding to 1% of the participants.

Normal ALT activity (<39 U/L) was noted in 88 individuals, encompassing 95.7% of the cohort, while activity ≥ 39 U/L were observed in 4 individuals, comprising 4.4% of the group. Similarly, AST activity was within the normal range (<51 U/L) in 91 individuals, representing 98.9% of the cohort, with only 1 individual (1.1%) exhibiting activity ≥ 51 U/L.

Normal total cholesterol concentration (<115 mg/dL) was observed in 3 individuals (3.3%), concentrations between 115 and 190 mg/dL in 76 individuals (84.4%), and concentrations exceeding 190 mg/dL in 11 individuals (12.2%). HDL concentration ≥ 40 mg/dL was found in 85 individuals (92.4%), while concentrations below 40 mg/dL were seen in 7 individuals (7.6%).

Regarding LDL concentration, 63 individuals (68.5%) had levels below 115 mg/dL, whereas 29 individuals (31.5%) had concentrations ≥ 115 mg/dL. TG concentration was ≤150 mg/dL in 85 individuals (93.4%) and >150 mg/dL in 6 individuals (6.6%).

Glucose concentration was <100 mg/dL in 87 individuals (94.6%), while ≥100 mg/dL was observed in 5 individuals (5.4%) (Table 6).

Furthermore, HOMA-IR value was ≤2 in 40 individuals (44.0%) and ≥2 in 51 individuals (56.0%) of the study population.

#### 3.2.1. ALT

The mean ALT activity in children at the first visit was 17.0 ± 6.5 U/L for girls and 20.9 ± 12.5 U/L for boys. This value differed significantly between sexes (*p* = 0.05).

#### 3.2.2. AST

The mean AST activity in children at the first visit was 25.4 ± 6.8 U/L for girls and 25.6 ± 6.7 U/L for boys. This value did not differ significantly between sexes (*p* = 0.4).

#### 3.2.3. Glucose

The mean glucose value in children at the first visit was 90.2 ± 5.9 mg/dL for girls and 92.2 ± 5.4 mg/dL for boys. This value differed significantly between sexes (*p* = 0.09).

#### 3.2.4. Cholesterol

The mean cholesterol concentration in children at the first visit was 166.3 ± 28.2 mg/dL for girls and 152.0 ± 25.1 mg/dL for boys. This value differed significantly between sexes (*p* = 0.01).

#### 3.2.5. HDL

The mean HDL concentration in children at the first visit was 54.3 ± 11.0 mg/dL for girls and 53.1 ± 10.2 mg/dL for boys. This value did not differ significantly between sexes (*p* = 0.6).

#### 3.2.6. LDL

The mean LDL concentration in children at the first visit was 109.1 ± 24.6 mg/dL for girls and 95.7 ± 25.9 mg/dL for boys. This value differed significantly between sexes (*p* = 0.01).

#### 3.2.7. Insulin

The mean insulin value in children at the first visit was 10.2 ± 4.2 µIU/mL for girls and 10.4 ± 5.6 µIU/mL for boys. This value did not differ significantly between sexes (*p* = 0.9).

#### 3.2.8. HOMA-IR

The mean HOMA-IR value in children at the first visit was 2.3 ± 1.0 for girls and 2.4 ± 1.3 for boys. This value did not differ significantly between sexes (*p* = 0.7). The mean HOMA-IR value in children at the follow-up visit was 3.38 ± 1.67 for girls and 2.2 ± 1.1 for boys. This value did not differ significantly between sexes (*p* = 0.2).

#### 3.2.9. Lead

The average BLL among all children was 3.8 ± 2.30 µg/dL. For girls, the mean BLL was 3.66 ± 2.28 µg/dL, showing no statistically significant difference compared to boys, whose mean blood lead level was 3.99 ± 2.35 µg/dL (*p* = 0.4466).

Among children with normal glycemia (<100 mg/dL), the BLL averaged 4.1 ± 2.4 µg/dL, with no statistically significant difference compared to those with abnormal glycemia (≥100 mg/dL), where the mean was 4.4 ± 2.7 µg/dL (*p* = 0.7969).

In children with a HOMA-IR value ≤ 2, the BLL averaged 4.4 ± 2.5 µg/dL, showing no statistically significant difference (*p* = 0.4) from those with a HOMA-IR value > 2, where the mean was 4.0 ± 2.3 µg/dL.

For children with normal cholesterol concentration (≤190 mg/dL), the BLL averaged 4.2 ± 2.5 µg/dL, while for those with elevated cholesterol concentration (≥190 mg/dL), it was 3.8 ± 2.2 µg/dL, with no statistically significant difference (*p* = 0.6).

Among children with normal HDL concentration (≥40 mg/dL), the BLL averaged 2.0 ± 1.6 µg/dL, significantly lower than in those with abnormal HDL concentration (<40 mg/dL), where it was 4.24 ± 2.4 µg/dL (*p* < 0.05).

The BLL in children with normal LDL concentration (<110 mg/dL) averaged 4.3 ± 2.6 µg/dL, not statistically significantly higher than in those with elevated LDL concentration (≥130 mg/dL), where it was 3.7 ± 2.1 µg/dL (*p* = 0.4).

For children with normal TG concentration (<75 mg/dL), the mean BLL was statistically significantly higher (*p* < 0.01) at 4.23 ± 2.4 µg/dL compared to those with elevated TG concentration (≥100 mg/dL), where it was 1.3 ± 0.5 µg/dL.

### 3.3. Dependencies between Evaluated Parameters

The aim of the further statistical analysis was to assess the dependencies between the determined parameters and the blood lead levels in the examined children.

The average systolic blood pressure in the group of all children with blood lead levels ≤5 µg/dL was 101.92 ± 14.06 mmHg. The average systolic blood pressure in children with blood lead levels >5 µg/dL was 106.25 ± 10.99, but the observed difference was statistically insignificant (*p* = 0.1201) (Table 7).

The absolute fat tissue content in the group of all children with blood lead levels ≤ 5 µg/dL was 25.4 ± 2.92 for girls and 18.03 ± 5.94 for boys. Meanwhile, in children with blood lead levels > 5 µg/dL, it was 23.76 ± 5.63 for girls and 15.67 ± 4.86 for boys, with statistically insignificant differences (respectively: *p* = 0.1330; *p* = 0.1829) (Table S1 and Table 8).

Average body weight in the group of children with BLLs ≤ 5 µg/dL, considering all participants, was 38.78 ± 6.58 kg; in the group of girls, it was 38.41 ± 5.54 kg, and in the group of boys, it was 39.31 ± 7.88 kg. In the group of children with BLLs > 5 µg/dL, the average body weight was 38.35 ± 4.67 kg, with girls at 38.11 ± 4.78 kg and boys at 38.63 ± 4.7 kg. The observed differences were statistically insignificant for both sexes (respectively: *p* = 0.9524 and *p* = 0.9136) (Tables S2–S4).

The average fat tissue mass in children with BLLs ≤ 5 µg/dL was 8.62 ± 3.19 kg, considering all participants. For girls, it was 9.63 ± 2.26 kg, and for boys, it was 7.21 ± 3.77 kg. Considering all children with BLLs > 5 µg/dL, the average fat tissue mass was 7.53 ± 3.09 kg. For girls, it was 8.94 ± 2.86 kg, and for boys, it was 5.93 ± 2.58 kg, with statistically insignificant differences (respectively: *p* = 0.6067 and *p* = 0.2987) (Tables S2–S4).

Muscle mass in the group of children with BLLs ≤ 5 µg/dL was 27.18 ± 4.25 kg. For girls, it was 25.79 ± 3.37 kg, and for boys, it was 29.15 ± 4.63 kg. In all children with BLLs > 5 µg/dL, the muscle mass was 27.66 ± 3.02 kg, with girls at 26.00 ± 2.24 kg and boys at 29.53 ± 2.72 kg. These values did not differ significantly between sexes (respectively: *p* = 0.6963 and *p* = 0.0.3762) (Tables S2–S4).

The ALT activity in the group of children with BLLs ≤ 5 µg/dL was 17.4 ± 6.9 for girls and 17.5 ± 8.4 for boys. In children with BLLs > 5 µg/dL, girls had a mean ALT activity of 15.7 ± 5.8, while boys had a mean ALT activity of 25.7 ± 18.87. These values did not differ significantly statistically between the sexes (respectively: *p* = 0.5 and *p* = 0.2).

### 3.4. Correlations

Considering all the children included in this study (n = 136), the analysis revealed a statistically significant positive correlation between blood lead levels and mean systolic blood pressure (r = 0.26; *p* = 0.0076) as well as mean diastolic blood pressure (r = 0.19; *p* = 0.0489). Additionally, a negative correlation was observed between blood lead levels and absolute fat tissue content (r = −0.19, *p* = 0.0484) as well as triglyceride levels (r = −0.26, *p* = 0.0241) (Table S5, Table 9 and Table 10).

The backward stepwise multiple regression analysis for the entire study group (n = 87) with fat mass as the dependent variable yielded an adjusted R^2^ = 0.094. Among the included biochemical factors, only insulin demonstrated a statistically significant relationship (β = 0.36; *p* = 0.002) with fat mass. The inclusion of Pb results as explanatory factors did not significantly alter the final R^2^ = 0.107. Once again, insulin emerged as the sole factor showing a statistically significant relationship (β = 0.436; *p* = 0.003) with fat mass.

A slightly improved fit of the statistical model was achieved to explain fat tissue mass (R^2^ = 0.198). Similarly to the previous analysis, only insulin exhibited a statistically significant relationship (β = 0.302; *p* = 0.000). The inclusion of Pb weakened the mutual dependence (R^2^ = 0.171) of fat tissue mass on the included factors while retaining the statistical significance of insulin (β = 0.365; *p* = 0.000).

Regression analysis for fat-free mass indicates that biochemical measurements explain 23% (R^2^ = 0.228) of the variation in this parameter. Notably, three examined parameters (ALT, AST, and insulin) showed statistical significance (respectively: β = 0.133, *p* = 0.017; β = −0.215, *p* = 0.003; β = 0.323, *p* = 0.003). After considering Pb in the analysis, a drastic decrease in R^2^ to 0.0647 was observed. Insulin maintained its statistical significance with β = 0.274; *p* = 0.044.

## 4. Discussion

### 4.1. Lead in Children’s Blood

The results of our research conducted among obese children living in the north-western region of Poland showed that a large proportion of the participants had BLLs above the WHO threshold for lead. BLLs ≤ 5 µg/dL (considered safe for children and pregnant women) were found in over 70% of the participants, with BLLs in the range of 5.01–10.00 µg/dL in over 26% of the children, and concentrations > 10 µg/dL (considered toxic threshold for adults) in nearly 2% of the children.

Lead toxicity in children remains a significant health concern, primarily due to environmental exposure [18]. In developed countries, growing awareness of lead’s impact on the environment and human health has driven efforts to reduce its use in products such as fuels, paints, ceramics, batteries, solders, and various consumer goods (e.g., artificial sports fields made from nylon or nylon/polyethylene blends, plastic toys, and jewelry) [18]. A persistent source of Pb exposure in early childhood, including in Poland, is lead-contaminated household dust (from deteriorating lead-based paint and lead-contaminated soil) and tap water contaminated by lead pipes. Current sources of Pb in the atmosphere include smelting, mining and ore processing, lead-acid battery production, and coal combustion, such as in power generation [19].

The blood lead levels (BLLs) determined in our study were surprisingly high. Unfortunately, Poland has not conducted comprehensive cohort studies on BLLs for many years, especially in the areas covered by our research. The UNICEF report “Places and Spaces: The Impact of Environment on Children’s Welfare” compared 39 European Union countries and members of the Organization for Economic Cooperation and Development (OECD). It examined the impact of environmental factors on children’s well-being and how countries are dealing with eliminating harmful factors that may negatively affect the health and development of the youngest. According to UNICEF data, over 260,000 children in Poland (3.6% of all children) have elevated BLLs (>5 µg/dL). A similar situation exists in nine of the world’s wealthiest countries, where more than 1 in 20 children have excessively high levels of this element in their bodies. This is primarily due to air, water, and food pollution, with Pb in the environment being one of the causes of serious disorders and diseases in children. Although it is currently believed that there is no safe threshold for Pb exposure and harmful effects can occur even at very low BLLs [11], in all OECD Charter countries, at least 1 in 100 children had elevated BLLs [20]. In most countries, this percentage is higher than 1 in 50 [20]. The results of our research indicate an even higher percentage of children with elevated BLLs than the one indicated in the cited report [20,21].

In Poland, a study conducted by [22] among 6-year-old children living in Kraków between 1997 and 2004 showed that 5% of the participants had BLLs significantly exceeding 10 µg/dL (BLL range 1.9–32.7 µg/dL). On the other hand, ref. [23] in a study of newborns born between 2001 and 2004 to mothers living in the Kraków area, showed that over 90% of newborns had BLLs below 5 µg/dL (range 0.44–6.90 µg/dL). Conversely, ref. [24] in a study conducted among children aged 7–11 years living in major cities in Europe and Sweden between 2007 and 2008, showed slight differences in the mean value of BLL (Slovenia 1.34 µg/dL; Sweden 1.40 µg/dL; Czech Republic 1.55 µg/dL; Poland 1.63 µg/dL; Croatia 1.79 µg/dL; Slovakia 1.94 µg/dL). In the same study, the mean BLL in children from countries outside Europe was significantly higher (Ecuador 3.17 µg/dL and Morocco 7.10 µg/dL).

Similarly, a study conducted in the United States between 1999 and 2002 showed that 1.4% of children aged 1–5 years had BLLs ≥ 10 μg/dL [25]. However, in China, as many as 34% of children aged 1–5 years were found to have BLLs ≥ 10 μg/dL [26], while in South Africa, it was 78% [27], and in Bangladesh, it was 87% of children aged 4–12 years [28].

Children may be exposed to Pb from various sources. In their own homes, this may include cosmetics, peeling paints, and the dust they generate, toys, clothing, jewelry, kitchen utensils, and even plumbing [21]. Pb can enter food through soil or water [29], and long-term contamination with leaded gasoline can still be found in soils worldwide [30]. In the past, Pb was present in children’s products, such as enameled and painted toys. Currently, it can still be found in leaded ceramics (e.g., in Mexico), lead pellets used in hunting and fishing (which may be a source of exposure among children who consume game and game birds), and spices and herbs mixed with clay containing lead to increase weight or color (often produced in South Asia, China, but imported worldwide) [31,32]. Lead is also present in the dust on playgrounds, which are lined with material contaminated with Pb [33]. A significant source of exposure in African countries remains the illegal recycling of lead-acid batteries and electronic waste exposure [33].

### 4.2. The Blood Lead Levels and Blood Pressure in Children

The results of our research revealed a positive association between BLLs and average systolic and diastolic blood pressure in the studied children. It is already known that interactions between external environmental factors and internal factors can lead to dysfunction of the circulatory system and accelerate the development of cardiovascular diseases, including arterial hypertension. Genetic factors (such as polymorphisms of various genes), metabolic factors (involved in lipid, protein, and carbohydrate metabolism), vascular factors (hormonal, nitric oxide), inflammatory factors participating in coagulation and fibrinolysis processes, and external factors (such as chronic stress, cigarette smoking, excess sodium in the diet) may contribute to its development [34,35].

The external factors include exposures to xenobiotics, which can affect the circulatory system indirectly by influencing brain centers, the autonomic nervous system, the secretion of vasoactive mediators, and directly by acting toxically on cardiomyocytes and vascular wall cells, for example, by interacting with intracellular redox pathways (NF-kappaB, AP-1, p53) [36]. Heavy metals, including lead, have been confirmed to have hypertensive effects [37,38].

It has been shown that Pb, at higher concentrations, can induce renal hypertension of nephrotic origin. It can also cause a significant increase in blood pressure by affecting the kinin pathway and through the renin–angiotensin system, increasing angiotensin II levels [39,40,41]. However, Pb also induces arterial hypertension at low blood concentrations, where no toxic effects on organs are manifested [36,42]. The hypertensive effect of Pb has been demonstrated in numerous experimental and population studies [37,38,43].

A positive relationship between BLLs and systolic and/or diastolic blood pressure or the risk of hypertension has been shown in men in the Normative Aging Study [44], and in peri- and postmenopausal women [37]. In women with pregnancy-induced hypertension, the BLL was significantly higher than in normotensive pregnant women [43]. A study conducted in South Korea based on a representative sample of 11,979 adults between 2008 and 2013 suggested that the BLL was associated with higher blood pressure and an increased risk of hypertension [45]. Similarly, a population-based study involving 948 Brazilian adults aged 40 and older showed that BLL > 2.76 μg/dL was associated with an increase in diastolic blood pressure by 0.06 mm Hg compared to BLLs ≤ 1.32 µg/dL.

There are also studies that do not show a relationship between BLLs and arterial blood pressure or indicate a weak association [46,47]. From a meta-analysis including 58,518 individuals environmentally or occupationally exposed to Pb, taking into account various confounding factors (such as age, body mass index, alcohol consumption, and medication use), it follows that a doubling of BLLs is associated with an increase in systolic blood pressure by 1.0 mmHg and diastolic blood pressure by 0.6 mmHg [35].

Diverse results regarding the impact of Pb on blood pressure in population studies may arise from the observation that the development of hypertension is attributed to the pool of stored Pb, with less current exposure [42]. Therefore, the concentration of lead in bones may show a stronger association with arterial blood pressure values than BLLs [48], which may be particularly relevant in children. It can also be concluded that in the assessment of lead-induced hypertension, data from early stages of life, from prenatal life through lactation to early childhood, may be more relevant than the BLL [49,50,51].

A cohort study of 323 children from New Hampshire, USA conducted a study that prospectively examined the association between prenatal Pb exposure and blood pressure in early childhood, using measurements of Pb concentration in maternal toenails collected at two time points to assess different prenatal exposure periods. The study authors found that each doubling of Pb concentration in maternal toenails during the prenatal period, reflecting exposure from the perinatal period to the early prenatal period, was associated with an increase in systolic blood pressure in children at 5.5 years of age, and this correlation was stronger in boys [52].

However, the existing literature shows only limited data regarding the impact of the BLL on the development of arterial hypertension in overweight and obese children. Therefore, it seems that the results of our research are significant for understanding this relationship.

### 4.3. Blood Lead Levels and Absolute Fat Tissue Content and TG Concentration

In our study, we demonstrated a negative correlation between the BLL and absolute fat tissue content, as well as TG concentration. Previous research indicates that Pb acts proatherogenically, causing an increase in total cholesterol, LDL, triacylglycerols, and a decrease in HDL levels. However, there are also studies where chronic Pb exposure results in decreased total cholesterol and LDL levels, as well as increased HDL levels in humans [53].

In a cross-sectional study conducted among obese Mexicans, both men and women with a mean age of 20.3 ± 1.9 years, and a mean serum blood lead level (Pb-S) of 0.0982 ± 0.068 µg/dL, and overweight and obesity rates of 50.5% and 18.8%, respectively, an analysis of anthropometric and clinical variables allowed the authors to determine that Pb-S and the frequency of overweight and obesity increased in Pb-S tertiles. The mean Pb-S levels were 0.051 ± 0.035 µg/dL, 0.107 ± 0.067 µg/dL, and 0.151 ± 0.063 µg/dL, respectively, for individuals with normal weight, overweight, and obesity [54].

Furthermore, an analysis adjusted for age and sex showed that Pb-S was positively associated with BMI. These findings align with research demonstrating a positive association between the BLL and BMI and obesity in the East Asian population [55]. Long-term study results have also shown a positive association between childhood Pb levels in dentin and BMI [56].

In study of Wang et al. another study, maternal BLL was associated with the risk of overweight and obesity in children in a dose–response relationship, where mothers and children with overweight and obesity had the highest Pb levels compared to mothers and children with normal weight [57]. However, some studies showed a negative relationship [58,59] or no relationship between overweight, obesity, and lead levels in children [60] and adults [61].

In our study on children, we found a negative correlation between the blood lead level (BLL) and both fat tissue content and triglyceride concentration. However, no significant relationship was observed between Pb and BMI, which contrasts with findings from other researchers [55,56]. This discrepancy may stem from variations in the biological samples used for lead analysis (e.g., serum, plasma, urine, bones, hair, or other matrices), as well as differences in age and dietary habits. Given the limited data on blood lead levels and fat tissue content in children, further research in this area is needed.

### 4.4. Blood Lead Levels and Insulin

The study we conducted primarily focused on the potential impact of Pb on the occurrence of overweight and its metabolic complications in children. We noted that among the biochemical factors considered, only insulin showed a statistically significant correlation (β = 0.36; *p* = 0.002) with body fat mass. This result suggests that early carbohydrate metabolism disorders (as impaired glucose tolerance and insulin resistance) in overweight children involve decreased peripheral tissue insulin sensitivity. With an increase in fat tissue mass, insulin resistance increases, and disorders such as impaired glucose tolerance or impaired fasting glucose may occur later. Although we selected 8-year-old children for analysis, not all of them likely had a pre-pubertal status. In our study group, girls and boys did not differ in mean age or BMI during the first visit (Table 4), but they exhibit differences in fasting glucose levels. This variation can be attributed to differences in body composition (Table 5). Girls have a statistically significant higher percentage of body fat, which could explain the elevated glucose levels. Eight-year-old girls may have already started their puberty, explaining the differences in fat tissue content between them and 8-year-old boys observed in our study.

## 5. Limitation of This Study

Our paper draws attention to the impact of Pb on the development of obesity in children exposed to environmental pollution. The strong point of this paper is the homogeneous study group comprising children of identical age from a single geographic region. Moreover, while the influence of environmental toxins on adults has been extensively explored, this study delves into pediatric populations, which have yet to receive com-prehensive scrutiny within the scientific literature. On the other hand, such a specific group of children studied is also a certain limitation and it would be worthwhile to extend our research to children of different ages.

## 6. Conclusions

Lead exposure may significantly contribute to the development of hypertension, impaired glucose tolerance and insulin resistance in overweight and obese children. It is essential to implement multidirectional actions to increase awareness of the harmful effects of xenobiotic exposure, including Pb, in order to prevent early-life exposure.

## Figures and Tables

**Table 1:** Lipid norms based on the American Academy of Pediatrics (AAP) guidelines [17].

Lipids	Age	Values (mg/dL)		
		Optimal	Borderline	High
Total cholesterol		<170	170–199	≥200
LDL		<110	110–129	≥130
HDL	<10 years	>40	30–40	
Triglycerides	<10 years	<75	75–99	≥100

**Table 2:** Auxological data.

Parameter	*n*	$\bar{x}$	SD	Min	Max	M
age [years]	129	8.27	0.8	7.1	10.7	8.1
body weight [kg]	135	38.5	6.2	25.5	59.6	37.7
SDS of body weight	129	4.0	2.2	0.3	18.6	3.7
body height [cm]	135	133.3	6.6	116	156	133
SDS of body height	129	0.8	1.1	−3.3	3.4	0.8
BMI [kg/m^2^]	135	21.5	2.2	15.4	31.4	21.1
SDS of BMI	129	4.5	1.8	−0.3	13.7	4.1
systolic blood pressure [mmHg]	134	104.2	8.8	80	138	104
diastolic blood pressure [mmHg]	134	67.5	7.3	51	84	67

*n*—number of samples, $\bar{x}$—arithmetic mean, SD—standard deviation, and M—median.

**Table 3:** Body composition.

Parameter	*n*	$\bar{x}$	SD	Min	Max	M
body fat content [%]	135	22.0	6.0	3	33	24
fat tissue mass [kg]	135	8.4	3.2	0	17	8
fat-free mass [kg]	135	29.3	4.3	21	44	29
water content [%]	135	21.0	3.2	15	32	21
muscle mass [kg]	135	27.1	4.1	19	41	27

*n*—number of samples, $\bar{x}$—arithmetic mean, SD—standard deviation, and M—median.

**Table 4:** Auxological data by sex.

Parameter	Girls			Boys			*p*
	*n*	$\bar{x}$	SD	*n*	$\bar{x}$	SD	
age [years]	73	8.3	0.7	56	8.3	0.8	0.0
body weight [kg]	79	37.9	5.5	56	29.5	7.1	0.1
SDS of body weight	73	3.8	1.8	56	4.2	2.7	0.3
body height [cm]	79	132.6	6.2	56	134.3	7.0	0.1
SDS of body height	73	0.7	1.2	56	0.9	1.1	0.4
BMI [kg/m^2^]	79	21.3	1.9	56	21.7	2.7	0.3
SDS of BMI	73	4.4	1.4	56	4.7	2.2	0.3
systolic blood pressure [mmHg]	79	104.5	8.9	56	102.1	16.3	0.3
diastolic blood pressure [mmHg]	79	68.8	6.9	56	64.4	11.5	<0.01

*n*—number of samples, $\bar{x}$—arithmetic mean, SD—standard deviation, and *p*—significance level (Student’s *t*-test).

**Table 5:** Body composition by sex.

Parameter	Girls			Boys			*p*
	*n*	$\bar{x}$	SD	*n*	$\bar{x}$	SD	
body fat content [%]	79	25.1	3.8	56	17.6	5.7	<0.001
fat tissue mass [kg]	79	9.4	2.5	56	7.0	3.56	<0.001
fat-free mass [kg]	79	27.7	3.6	56	31.7	4.2	<0.001
water content [%]	79	19.7	2.5	56	22.7	3.0	<0.001
muscle mass [kg]	79	25.4	3.8	56	29.5	4.0	<0.001

*n*—number of samples, $\bar{x}$—arithmetic mean, SD—standard deviation, and *p*—significance level (Student’s *t*-test).

**Table 6:** Biochemical data.

Parameter	*n*	$\bar{x}$	SD	Min.	Max.	Median
blood lead levels [µg/dL]	115	3.8	2.3	0.1	10.5	3.6
ALT [U/L]	92	18.4	9.3	5	70	15
AST [U/L]	92	25.8	6.8	13	63	25
glucose [mg/dL]	92	90.9	5.8	75.9	106.2	90.8
glucose [mmol/L]	92	5.1	0.3	4.2	5.9	5.0
total cholesterol [mg/dL]	90	160.9	27.8	98.6	258	159.6
HDL [mg/dL]	92	53.9	10.7	33.4	86.3	53.4
LDL [mg/dL]	92	104.1	25.8	32.7	197.3	101.4
TG [mg/dL]	91	75.6	38.1	22.6	213	67.3
insulin [µIU/mL]	91	10.3	4.8	2.5	32.1	9.4
HOMA IR	91	2.3	1.1	0.5	6.7	2.1

*n*—number of samples, $\bar{x}$—arithmetic mean, SD—standard deviation.

**Table 7:** Biochemical data by sex.

Parameter	Girls			Boys			*p*
	*n*	$\bar{x}$	SD	*n*	$\bar{x}$	SD	
blood lead levels [µg/dL]	66	3.7	2.3	49	4.0	2.4	0.4
ALT [U/L]	58	17.0	6.5	49	20.9	12.5	0.05
AST [U/L]	58	25.4	6.8	49	25.6	6.7	0.4
glucose [mg/dL]	58	90.2	5.9	49	92.2	5.4	0.09
total cholesterol [mg/dL]	56	166.3	28.2	49	152.0	25.1	0.01
HDL [mg/dL]	58	54.3	11.0	49	53.1	10.2	0.6
LDL [mg/dL]	58	109.1	24.6	49	95.7	25.9	0.01
TG [mg/dL]	57	76.7	32.9	49	74.0	46.0	0.7
insulin [uIU/mL]	57	10.2	4.2	49	10.4	5.6	0.9
HOMA IR	57	2.3	0.98	49	2.4	1.3	0.7

*n*—number of samples, $\bar{x}$—arithmetic mean, SD—standard deviation, and *p*—significance level (Student’s *t*-test).

**Table 8:** Body composition in boys in relation to the whole-blood lead concentration.

Parameter	Pb ≤ 5 µg/dL (n = 34)		Pb > 5 µg/dL (n = 15)		*p*
	$\bar{x}$	SD	$\bar{x}$	SD	
body weight [kg]	39.3	7.9	38.6	4.7	0.8
BMI [kg/m^2]^	21.8	3.0	21.3	1.7	0.6
systolic blood pressure [mmHg]	101.5	6.9	107.3	11.6	<0.05
diastolic blood pressure [mmHg]	64.7	6.8	65.1	7.4	0.8
fat tissue content [%]	18.0	5.9	15.7	4.9	0.2
fat tissue mass [kg]	7.2	3.8	5.9	2.6	0.2
fat-free mass [kg]	31.3	4.8	31.7	2.9	0.8
water content [%]	22.4	3.5	22.9	2.0	0.6
muscle mass [kg]	29.2	4.6	29.5	2.7	0.8

*p*—significance level (Student’s *t*-test).

**Table 9:** The correlation between anthropometric and biochemical measurements and the whole-blood lead levels.

Parameter	Blood Lead Level [µg/dL]		
	n	r	*p*
body weight [kg]	114	0.01	0.9
body height [cm]	114	0.04	0.7
BMI [kg/m^2^]	114	−0.02	0.9
systolic blood pressure [mmHg]	113	0.24	0.01
diastolic blood pressure [mmHg]	113	0.19	0.04
fat tissue content [%]	114	−0.20	0.03
fat tissue mass [kg]	114	−0.14	0.1
fat-free mass [kg]	114	0.11	0.2
water content [%]	114	0.13	0.2
muscle weight [kg]	114	0.12	0.2
ALT [U/L]	78	0.05	0.7
AST [U/L]	78	−0.05	0.7
glucose [mg/dL]	78	−0.05	0.7
cholesterol [mg/dL]	76	−0.08	0.5
HDL [mg/dL]	78	0.11	0.3
LDL [mg/dL]	78	−0.08	0.5
TG [mg/dL]	77	−0.26	0.02
insulin [µIU/mL]	77	−0.13	0.3
HOMA IR	77	−0.12	0.3

**Table 10:** Multidimensional correlation between anthropometric and biochemical measurements and lead levels.

Parameter	Blood Lead Level [µg/dL]		
	n	r	*p*
body weight [kg]	110	−0.02	0.8
body height [cm]	110	0.00	0.9
BMI [kg/m^2^]	110	−0.02	0.9
systolic blood pressure [mmHg]	109	0.26	<0.01
diastolic blood pressure [mmHg]	109	0.19	<0.05
fat tissue content [%]	110	−0.19	<0.05
fat tissue mass [kg]	110	−0.14	0.1
fat-free mass [kg]	110	0.06	0.5
water content [%]	110	0.08	0.4
muscle weight [kg]	110	0.06	0.5
ALT [U/L]	76	0.03	0.8
AST [U/L]	76	−0.09	0.5
glucose [mg/dL]	78	−0.07	0.5
cholesterol [mg/dL]	74	−0.06	0.6
HDL [mg/dL]	76	0.12	0.3
LDL [mg/dL]	76	−0.06	0.6
TG [mg/dL]	75	−0.26	<0.05
insulin [µIU/mL]	75	−0.13	0.3
HOMA IR	75	−0.13	0.3

## Data Availability

The data presented in this study are available in article and Supplementary Materials.

## Supplementary Materials

The following supporting information can be downloaded at: https://www.mdpi.com/article/10.3390/metabo14100540/s1, Table S1: Body composition in relation to the whole blood lead concentration; Table S2: Body composition in girls in relation to the whole blood lead concentration; Table S3: Biochemical data for all studied children in relation to the whole blood lead levels; Table S4: Biochemical data in boys in relation to whole blood lead levels; Table S5: Biochemical data for girls in relation to whole blood lead levels.
