# Supplementary material for: The Assessment of Anthropometric Measures and Changes in Selected Biochemical Parameters in Obese Children in Relation to Blood Lead Level
Source: Metabolites. 2024 Oct 9;14(10):540. doi: 10.3390/metabo14100540 (PMC11509403; doi:10.3390/metabo14100540)
Supplement: Supplementary file 1 [file metabolites-14-00540-s001.zip › metabolites-3195889-supplementary.pdf]

Table S1. Body composition in relation to the whole blood lead concentration.

Parameter	Pb≤5 ug/dl (n=82)		Pb>5 ug/dl (n=32)		p
	$\bar{x}$	SD	$\bar{x}$	SD	
body weight [kg]	38.8	6.6	38.3	4.7	0.7
BMI [kg/m2]	21.6	2.5	21.4	1.6	0.7
systolic blood pressure [mmHg]	101.9	14.1	106.3	11.0	0.1
diastolic blood pressure [mmHg]	65.9	10.0	67.6	7.8	0.4
fat tissue content [%]	22.3	5.7	20.0	6.6	0.1
fat tissue mass [kg]	8.6	3.2	7.5	3.1	0.1
fat-free mass [kg]	29.4	4.5	29.8	3.1	0.6
water content [%]	20.9	3.2	21.4	2.3	0.5
muscle mass [kg]	27.2	4.3	27.7	3.0	0.6

p – significance level (Student's t-test).

Table S2. Body composition in girls in relation to the whole blood lead concentration.

Parameter	Pb<5 ug/dl (n=48)		Pb>5 ug/dl (n=17)		p
	$\bar{x}$	SD	$\bar{x}$	SD	
body weight [kg]	38.4	5.5	38.	4.8	0.8
BMI [kg/m2]	21.4	2	21.5	1.5	0.9
systolic blood pressure [mmHg]	104.3	9.0	105.4	10.7	0.7
diastolic blood pressure [mmHg]	68.0	6.5	69.8	7.6	0.4
fat tissue content [%]	25.4	2.9	23.8	5.6	0.1
fat tissue mass [kg]	9.6	2.3	8.9	2.9	0.3
fat-free mass [kg]	28.0	3.7	28.2	2.4	0.8
water content [%]	19.9	2.6	20.1	1.8	0.8
muscle mass [kg]	25.8	3.4	26.0	2.2	0.8

p – significance level (Student's t-test).

Table S3. Biochemical data for all studied children in relation to the whole blood lead levels.

Parameter	Pb<5 μg/dl			Pb>5 μg/dl			p
	n	$\bar{x}$	SD	n	$\bar{x}$	SD	
ALT [U/l]	53	17.4	7.31	25	19.7	13.3	0.3
AST [U/l]	53	25.5	5.22	25	24.8	6.9	0.6
glucose [mg/dl]	53	91.4	4.0	25	91.2	6.7	0.9
cholesterol [mg/dl]	51	160.7	26.7	25	161.0	32.0	0.9
HDL [mg/dl]	53	53.4	9.1	25	56.1	12.0	0.3
LDL [mg/dl]	53	104.0	26.3	25	103.3	28.8	0.9
TG [mg/dl]	53	78.2	49.9	24	69.3	26.8	0.3
insulin [uIU/ml]	52	10.3	3.9	25	9.15	4.0	0.3
HOMA IR	52	2.34	0.99	25	2.09	1.0	0.3

p – significance level (Student's t-test).

Table S4. Biochemical data in boys in relation to whole blood lead levels.

Parameter	Pb<5 ug/dl (n=17)		Pb>5 ug/dl (n=10)		p
	$\bar{x}$	SD	$\bar{x}$	SD	
ALT [U/l]	17.5	8.4	25.7	18.9	0.1
AST [U/l]	26.4	6.7	27.3	8.3	0.8
glucose [mg/dl]	93.3	4.9	92.9	6.8	0.9
cholesterol [mg/dl]	154.6	26.7	150.7	16.1	0.7
HDL [mg/dl]	52.2	9.1	56.7	12.2	0.3
LDL [mg/dl]	97.8	26.3	93.8	17.3	0.7
TG [mg/dl]	76.5	49.9	65.4	29.5	0.5
insulin [μIU/ml]	10.3	3.9	7.9	4.1	0.1
HOMA IR	2.4	1.0	1.9	1.1	0.2

p – significance level (Student's t-test).

Table S5. Biochemical data for girls in relation to whole blood lead levels.

Parameter	Pb<5 ug/dl			Pb>5 ug/dl			p
	n	$\bar{x}$	SD	n	$\bar{x}$	SD	
ALT [U/l]	36	17.4	6.9	15	15.7	5.8	0.4
AST [U/l]	36	25.1	4.4	15	23.2	5.4	0.2
glucose [mg/dl]	36	90.5	5.8	15	90.0	6.6	0.8
cholesterol [mg/dl]	34	163.8	21.0	15	168.0	38.3	0.6
HDL [mg/dl]	36	54.0	11.0	15	55.7	12.2	0.6
LDL [mg/dl]	36	107.0	18.3	15	109.6	33.5	0.7
TG [mg/dl]	36	79.0	36.9	14	72.0	25.4	0.5
insulin [μIU/ml]	35	10.3	4.5	15	10.0	3.7	0.8
HOMA IR	35	2.3	1.0	15	2.3	0.9	0.8

p – significance level (Student's t-test).
